# Female Medical Aid to the Women of India

**Published:** 1887-11-26

**Authors:** 


### FEMALE MEDICAL AID TO THE WOMEN OF INDIA.

By the Countess of Dufferin, Lady President of the National Association.

In the early part of this year you were good enough to publish a letter in which I appealed to Members of the National Association and to all others interested in its work "to join me in making some special effort to commemorate Her Majesty the Queen-Empress' Jubilee, and at the same time to benefit those Indian women in whose welfare the Queen takes so great and personal an interest."

It is unnecessary for me to repeat the arrangements made to carry out this suggestion; but now that the collection is closed, I shall be glad if you will allow me, through the columns of your paper, to inform those who interested themselves in the matter, of the success of the effort made, and to give a few particulars with regard to the sums collected. On the 15th of October, on which day we were obliged to close the list of "Jubilee" subscriptions and donations, we had received Rs. 4,78,465 in India, and £1,770 in England. Our Jubilee collection therefore exceeds five lakhs.

Of this sum 3¼ lakhs were received in large donations, the remainder being the aggregate of smaller subscriptions on cards. Most of the branches of the Association took an active part in this collection, and Rs. 75,925 has been paid over to them, according to the rules laid down in my letter of the 1st of January.

To all donors of large sums and to the collectors of smaller ones I have sent receipts, and I have therefore, in some way, been able to acknowledge their kindness, and to express my appreciation of their generosity. I have, however, still to thank the tens of thousands of persons whose subscriptions, ranging from one anna to Rs. 100, have so greatly swelled this Jubilee collection, and whose gifts, appearing on other people's cards, have as yet received no personal recognition. I take this opportunity of doing so most heartily.

The list of donors and collectors is now being prepared for transmission to the Queen-Empress, and the same list, with the address forwarded to Her Majesty, will, for the information of subscribers, be published in the Report of the National Association in January, 1888. And here I must add that although this letter refers only to the "Jubilee collection" and to the money which has actually passed through my hands, it would be incomplete did it not contain at least a passing allusion to the well-directed efforts and to the large sums which are being spent in various parts of India upon female hospitals, and other works in connection with the National Association, and in commemoration of the Queen-Empress' Jubilee. Her Majesty's attention will be drawn to these, and detailed accounts of them will also appear in the Annual Report.

The system of collection by cards inaugurated on this occasion has certainly been successful. It has proved that vast numbers of persons are interesting themselves in the work of the Association, and it has shown that by a very simple organisation a lakh and a half can be collected in small sums such as thousands of persons are both able and willing to give. I hope therefore to continue it for the benefit of the Central Fund, though I cannot after this year undertake to receive money for the branches.

The Central Committee have now been enabled to invest the five lakhs which they were anxious to lay by as an Endowment Fund. The branches are likewise endeavouring to invest money so as to ensure the continuity of the work of the National Association. We trust therefore, that although the next year affords no special occasion for liberality, the progress already made in carrying out the objects of the Association, and the assurance of stability given to it by the possession of a certain, though a comparatively small income, will encourage all who are interested in the welfare of the women of India to make still further efforts on their behalf, and to give us the means of more quickly relieving their sufferings, and of supplying them with the medical aid which they so greatly need.

Viceregal Lodge, Simla, October 25th.

